# Immunoinformatics for Novel Multi-Epitope Vaccine Development in Canine Parvovirus Infections

**DOI:** 10.3390/biomedicines11082180

**Published:** 2023-08-02

**Authors:** Bashudeb Paul, Jahangir Alam, Mridha Md. Kamal Hossain, Syeda Farjana Hoque, Md. Nazmul Islam Bappy, Hafsa Akter, Nadim Ahmed, Margia Akter, Mohammad Ali Zinnah, Shobhan Das, Md. Mukthar Mia, Md. Shafiullah Parvej, Sonjoy Sarkar, Hiren Ghosh, Mahmudul Hasan, Hossam M. Ashour, Md. Masudur Rahman

**Affiliations:** 1 Department of Anatomy and Histology, Faculty of Veterinary, Animal and Biomedical Sciences, Sylhet Agricultural University, Sylhet 3100, Bangladesh; 2 Animal Biotechnology Division, National Institute of Biotechnology, Dhaka 1349, Bangladesh; 3 Department of Biological Sciences, University of Cincinnati, Cincinnati, OH 45221, USA; 4 Department of Animal and Fish Biotechnology, Faculty of Biotechnology and Genetic Engineering, Sylhet Agricultural University, Sylhet 3100, Bangladesh; 5 Department of Biochemistry and Chemistry, Faculty of Biotechnology and Genetic Engineering, Sylhet Agricultural University, Sylhet 3100, Bangladesh; 6 Faculty of Biotechnology and Genetic Engineering, Sylhet Agricultural University, Sylhet 3100, Bangladesh; 7 Department of Pathology, Faculty of Veterinary, Animal and Biomedical Sciences, Sylhet Agricultural University, Sylhet 3100, Bangladesh; 8 Department of Microbiology and Public Health, Bangabandhu Sheikh Mujibur Rahman Agricultural University, Gazipur 1706, Bangladesh; 9 Jiann-Ping Hsu College of Public Health, Georgia Southern University, Statesboro, GA 30458, USA; 10 Department of Poultry Science, Faculty of Veterinary, Animal and Biomedical Sciences, Sylhet Agricultural University, Sylhet 3100, Bangladesh; 11 Faculty of Veterinary and Animal Sciences, Gono University, Savar 1344, Bangladesh; 12 Institute for Infection Prevention and Hospital Epidemiology, Medical Center, University of Freiburg, 79106 Freiburg, Germany; 13 Department of Pharmaceuticals and Industrial Biotechnology, Faculty of Biotechnology and Genetic Engineering, Sylhet Agricultural University, Sylhet 3100, Bangladesh; 14 Department of Integrative Biology, College of Arts and Sciences, University of South Florida, St. Petersburg, FL 33701, USA; 15 ABEx Bio-Research Center, Dhaka 1230, Bangladesh

**Keywords:** canine parvovirus, capsid protein, epitope, molecular docking, vaccine design

## Abstract

Canine parvovirus (CPV-2) is one of the most important pathogens of dogs of all ages, causing pandemic infections that are characterized by fatal hemorrhagic enteritis. The CPV-2 vaccine is recommended as a core vaccine for pet animals. Despite the intensive practice of active immunization, CPV-2 remains a global threat. In this study, a multi-epitope vaccine against CPV-2 was designed, targeting the highly conserved capsid protein (VP2) via in silico approaches. Several immunoinformatics methods, such as epitope screening, molecular docking, and simulation were used to design a potential vaccine construct. The partial protein sequences of the VP2 gene of CPV-2 and protein sequences retrieved from the NCBI were screened to predict highly antigenic proteins through antigenicity, trans-membrane-topology screening, an allergenicity assessment, and a toxicity analysis. Homologous VP2 protein sequences typically linked to the disease were identified using NCBI BLAST, in which four conserved regions were preferred. Overall, 10 epitopes, DPIGGKTGI, KEFDTDLKP, GTDPDDVQ, GGTNFGYIG, GTFYFDCKP, NRALGLPP, SGTPTN, LGLPPFLNSL, IGGKTG, and VPPVYPN, were selected from the conserved regions to design the vaccine construct. The molecular docking demonstrated the higher binding affinity of these epitopes with dog leukocyte antigen (DLA) molecules. The selected epitopes were linked with *Salmonella enterica* flagellin FliC adjuvants, along with the PADRE sequence, by GGS linkers to construct a vaccine candidate with 272 nucleotides. The codon adaptation and in silico cloning showed that the generated vaccine can be expressed by the *E. coli* strain, K12, and the sequence of the vaccine construct showed no similarities with dog protein. Our results suggest that the vaccine construct might be useful in preventing canine parvoviral enteritis (CPE) in dogs. Further in vitro and in vivo experiments are needed for the validation of the vaccine candidate.

## 1. Introduction

Canine parvoviral enteritis (CPE) has long been known as one of the most dreaded viral infections of canids, leading to vomiting and hemorrhagic diarrhea in dogs of all ages; however, it is much more pronounced in young juvenile puppies 2–3 weeks of age [1,2,3]. The etiological agent, the canine parvovirus-2 (CPV-2), belongs to the genus *Protoparvovirus*. The CPV-2 is a single-stranded negative-sense DNA virus (its length is ~5200 nucleotides and its diameter is ~25 nm), which is enclosed within an icosahedron capsid [4,5,6]. The viral DNA encodes two non-structural proteins (NS1 and NS2) that help to control viral replication and assembly, and two structural proteins, VP1 and VP2 [6,7]. The VP2 protein (64 kDa) is an NH2 terminally truncated form of VP1 (84 kDa) that constitutes about 90% of the viral capsid; it is highly antigenic and plays a vital role in determining host ranges and tissue tropism [8,9]. Mutations in the amino acid sequence of this VP2 protein have been reported, and the original CPV-2 appears to have evolved into three main antigenic variants (CPV-2a, CPV-2b, and CPV-2c) [10,11]. Mutations in CPV-2a and -2b were also identified in the VP2 gene at residue 297 (from serine (Ser) to alanine (Ala)) leading to the new CPV 2a and 2b variants. Mutations (5Gly, 267Tyr, 324Ile, and 370Arg) in the VP2 gene of CPV-2c, considered to be the Asian CPV-2c genotype, have also recently been reported in some Asian countries [12].

Since its emergence, CPE has been regarded as a highly contagious viral disease in pet dogs and dogs in shelters. Most importantly, it causes significant economic losses for breeding farms [10,11,12,13]. Vaccination is considered the most effective method to prevent and control the spread of parvoviral infection. The CPV-2 vaccine is recommended as the core vaccine for pet animals by the American Animal Hospital Association (AAHA) [14]. The vaccination is mainly based on the use of inactivated (killed) and modified live virus (MLV) vaccines. Although they are safer, inactivated vaccines are less effective in preventing subclinical CPV infection. The widely used MLV vaccines stimulate immune responses and induce strong, long-acting protection against viruses. However, CPV-2 remains a global threat to the canine population, even in vaccinated animals. Vaccination failure is one of the principal causes of the continuous circulation of the virus throughout the world and the generation of its variants [15]. A series of parvovirus-like strains (which probably evolved from MLV vaccine strains) were reported in diseased and vaccinated animals [16]. Therefore, there is a dire need to develop alternative vaccines against parvoviral infections in susceptible animals.

The latest computational approaches and accessibility to large volumes of sequence information of interest have attracted many researchers to accelerate the process of vaccine design [17]. In this study, a multi-epitope vaccine against canine parvovirus was designed by targeting the highly conserved capsid protein (VP2), which encompasses important B- and T-cell epitopes in the N-domain and loop-domain via immunoinformatic approaches. Because of the highly conserved nature of these proteins and their role in pathogenesis and the generation of the immune response, they can be potential candidates for epitope identification in the design of a vaccine construct that can protect the canine population from fatal canine parvoviral infections [18,19]. Such approaches were previously used to design multi-epitope vaccines against a variety of organisms, such as *Mycoplasma bovis*, *Mycoplasma hyopneumoniae*, *Leishmania donovani*, *Candida auris*, *Helicobacter pylori*, *Tropheryma whipplei*, *Klebsiella pneumonia*, *Elizabethkingia anopheles*, Hepatitis C virus, Dengue virus, Zika virus, FMD virus, and SARS-COV-2 [20,21,22,23,24,25,26,27,28,29,30,31,32]. Several multi-epitope-based vaccines developed using these immunoinformatics approaches have been reported to elicit immune responses and confer significant protection in laboratory animals [21,31].

The design of a vaccine candidate using immunoinformatics approaches reduces the time and cost of vaccine development [33]. In our study, several immunoinformatics approaches were used in the design of a potential vaccine construct against canine parvovirus. The highly antigenic epitopes were identified. The interactions of these epitopes with dog leukocyte antigen (DLA) molecules were examined. We conducted our assessments using molecular docking and performed a molecular-dynamics simulation. Using GGS linkers, we linked the antigenic epitopes with *Salmonella enterica* flagellin FliC adjuvants and incorporated the PADRE sequence. This allowed us to construct a potential vaccine candidate. A physico-chemical analysis was undertaken, and the secondary and tertiary structures of the engineered vaccine construct were predicted. Furthermore, the antigenicity and allergenic potential were assessed.

Using different web servers, the molecular docking and molecular-dynamics simulation of the designed vaccine construct against TLR-5 were performed. In addition, we performed in silico cloning to assess the feasibility of cloning and expressing the vaccine construct. The construct was predicted to be beneficial in protecting dogs from canine parvovirus infections. 

## 2. Materials and Methods

**Figure 1:**
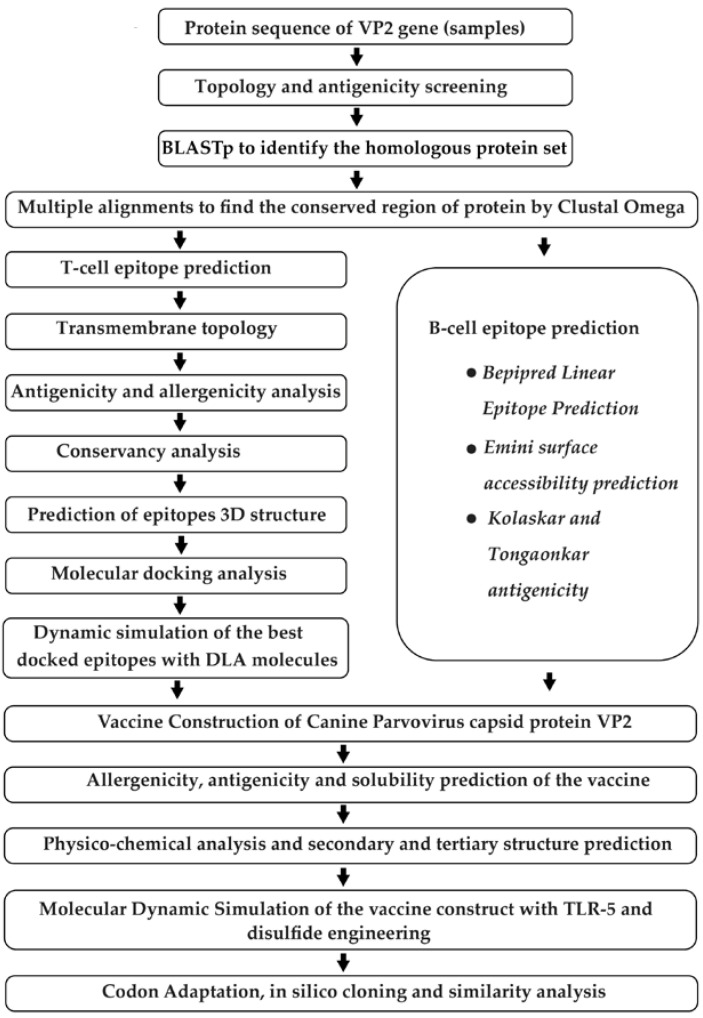
Schematic representation of the step-by-step phases used for designing the multi-epitope vaccine construct against canine parvovirus based on immunoinformatics techniques. The method starts with the retrieval of protein sequence followed by epitope prediction, secondary and tertiary structure of vaccine prediction, refining of molecular docking, and simulation. It ends with codon adaptation and in silico cloning.

Several steps were taken to develop a multi-epitope vaccine against the VP2 capsid proteins of CPV-2 (Figure 1).

### 2.1. Retrieval of VP2-Protein Sequences from the Corresponding Gene Sequences of Local CPV-2 Strain

The partial protein sequence of the VP2 gene of canine parvovirus was obtained (Genetic Analyzer 3130, Applied Biosystems, Waltham, MA, USA) using the dideoxy-chain-termination method after PCR amplification of the viral gene from rectal swabs of diseased dogs. The National Center for Biotechnology Information (NCBI) (Bethesda, MD, USA) proteomic database was also used for the selection and retrieval of VP2-protein sequences of canine parvovirus. 

### 2.2. Topology and Antigenicity Screening

The TMHMM Server v.2.0 (http://www.cbs.dtu.dk/services/TMHMM/, accessed on 18 January 2022) [34] and VaxiJen v.2.0 (http://www.ddgpharmfac.net/vaxijen/, accessed on 19 January 2022) [35] were used to predict the topology of each VP2 protein sequence and find the most potent antigenic proteins. Antigenic proteins with high antigenic scores were selected for further analyses.

### 2.3. Identification of Homologous Proteins and Analysis of Conserved Regions 

To begin, BLASTp was used to search for homologous VP2-protein sequences most commonly associated with the disease. Multiple sequence alignment (MSA) was performed using the ClustalOmega server (https://www.ebi.ac.uk/Tools/msa/clustalo/, accessed on 16 January 2022) [36] to identify conserved regions among the homologous sequences. This server is a revised form of the Clustal series of programs to perform MSA, and it can cover a large number (tens of thousands) of genome or protein sequences due to the use of the mBED algorithm to calculate guide trees. In addition, the asterisks on the conserved fragments facilitate identification of the conserved regions. The antigenicity and topology of the conserved region were revealed using the VaxiJen and TMHMM server. 

### 2.4. T-Cell Epitope Prediction, Trans-Membrane Topology Screening, and Antigenicity Analysis 

The conserved regions were used for T-cell-epitope enumeration via the Immune Epitope Database (IEDB) server (http://tools.iedb.org/main/tcell/, accessed on 21 January 2022) [37]. The IEDB server makes it possible to search for empirical data characterizing B-cell epitopes and T-cell epitopes. From this IEDB, the MHC-I prediction tool (http://tools.iedb.org/mhci/, accessed on 21 January 2022) was used to predict MHC-I binding [37]. Again, the TMHMM server was utilized for the prediction of the transmembrane topology of predicted MHC-I binding peptides followed by antigenicity scoring via the VaxiJen v2.0 server [34,35]. The most potent antigenic epitopes were selected and used for the subsequent analysis. 

### 2.5. Assessment of Allergenicity and Toxicity of T-Cell Epitopes 

Several bioinformatics tools, i.e., AllerTOP (http://www.ddgpharmfac.net/AllerTop/, accessed on 24 January 2022) [38], AllergenFP (http://www.ddgpharmfac.net/AllergenFP/, accessed on 24 January 2022) [39], and Allermatch (http://www.allermatch.org/allermatch.py/form, accessed on 24 January 2022) [40] were used. The non-allergenic epitopes were selected to assess the toxicity levels using the ToxinPred server (http://crdd.osdd.net/raghava/toxinpred/, accessed on 24 January 2022) [41].

### 2.6. Identification of B-Cell Epitopes

Three different algorithms, i.e., Bepipred Linear Epitope Prediction 2.0 [42], Emini surface accessibility prediction [43], and Kolaskar-and-Tongaonkar antigenicity scale [44], from IEDB (accessed on 25 January 2022), were used to identify the potential B-cell epitopes within conserved fragments of the VP2 proteins.

### 2.7. Selection of the Superior Epitopes and Their Conservancy Analysis 

Best T-cell and B-cell epitopes were scrutinized based on their antigenic score, toxicity, and allergenicity. This was followed by predicting the conservancy of these epitopes among the homologous strains using the conservancy-analysis tools by IEDB (http://tools.iedb.org/conservancy/, accessed on 26 January 2022) [37].

### 2.8. 3D-Structure Predictions of Superior T-Cell Epitopes and Docking at the Allele Level

Tertiary structures of the chosen epitopes were determined utilizing the PEP-FOLD 3.5 server (accessed on 27 January 2022) [45] via docking with the DLA alleles DLA-88*001:04 (PDB ID: 7CJQ) [46] and DLA-88*50801 (PDB ID: 5F1I) [46,47], as suggested in previous articles [48,49]. Docking was performed using the PatchDock server (accessed on 29 January 2022) to ensure the binding of epitopes with DLA [50]. The PatchDock algorithm sorts the Connolly dot-surface representation [51] of the molecules into concave, convex, and flat patches. Complementary patches were then matched and candidate transformations were generated. An additional scoring method that takes the geometric fit and the atomic desolvation energy into account was also applied to evaluate each potential transformation [52]. To eliminate redundant solutions, root mean square deviation (RMSD) clustering was applied to the candidate solutions. The solutions were sorted according to geometric-shape-complementarity score [53].

### 2.9. Vaccine Constructions

An epitope-based vaccine was constructed by combining all superior epitopes using a GGS linker. *Salmonella enterica* flagellin FliC was adjoined as an adjuvant to increase the efficiency of the constructed vaccine. The pan HLA DR-binding epitope (PADRE) sequence was also incorporated to increase the stability of the vaccine. The antigenic level of the constructed vaccine was predicted by the VaxiJen server and the allergenic potentiality of the vaccine molecule was checked using the AllerTOP server [38]. Protein-sol server (https://protein-sol.manchester.ac.uk, accessed on 9 February 2022) was utilized to predict the solubility of the vaccine construct [54].

### 2.10. Physico-Chemical Analysis and Structure Prediction of the Vaccine Construct

Stability and other physico-chemical features of the final vaccine construct were analyzed via the ProtParam tool (Biozentrum, University of Basel, Switzerland, at http://web.expasy.org/protparam/, accessed on 14 February 2022) [55]. The secondary structure was predicted using the PSIPRED server, which helped to predict the alpha helices, beta-sheets, and coils in the vaccine molecule [56]. Molecular modeling of the vaccine molecule was performed by the I-TASSER server [57], followed by the refinement of the structure by the GalaxyWeb server at the Computational Biology Lab in the Department of Biochemistry, Seoul National University (http://galaxy.seoklab.org/, accessed on 27 February 2022) [58]. After refinement, the best model was predicted by analyzing the model quality by using ERRAT and Procheck tools from the SAVES v6.0 server maintained by the National Health Institute, University of California, USA (http://services.mbi.ucla.edu/, accessed on 28 February 2022) [59,60]. 

### 2.11. Molecular Docking and Molecular Simulation against TLR-5

Molecular docking of the designed vaccine construct against TLR-5 was performed using the GalaxyTongDock server (accessed on 2 March 2022) [61], as TLR-5 was previously used in canine-vaccine prediction [62]. The 100 ns molecular dynamics simulation was carried out using the GROMACS (GROningen MAchine for Chemical Simulations, version 2020.6, GROMACS Development Team, Stockholm, Sweden) for GTFYFDCKP-DLA-88*001:04 complex, GGTNFGYIG-DLA-88*50801 complex, and vaccine construct–TLR5 complex since GTFYFDCKP-DLA-88*001:04 and GGTNFGYIG-DLA-88*50801 complexes exhibited the highest binding affinity for molecular docking [63]. The CHARMM36m force field was used for the simulation. Using the TIP3P water model, a water box was constructed, whose edges were 1 nm away from the protein surface. The systems were neutralized with the necessary ions. Following energy minimization, as well as isothermal–isochoric (NVT), and isobaric (NPT) equilibration of the system, a molecular dynamic simulation (100 ns) was performed under periodic boundary conditions and using 2 fs time-integration step. To analyze the trajectory data, a snapshot interval of 100 ps was used. After the simulation was completed, the rms, rmsf, gyrate, sasa, and hbond modules integrated within the GROMACS software (version 2020.6) were used to conduct the root-mean-square deviation (RMSD), root-mean-square fluctuation (RMSF), radius of gyration (Rg), and solvent-accessible surface area (SASA) analyses. The ggplot2 package in RStudio was utilized to generate the graphs for each of these analyses. All MD simulations were performed in the high-performance simulation stations running on Ubuntu 20.04.4 LTS operating system located at the Bioinformatics Division, National Institute of Biotechnology (Dhaka, Bangladesh).

### 2.12. Disulfide Engineering of the Designed Vaccine

Disulfide engineering of the predicted model was performed to replace the suitable amino acids with cysteine in the highly mobile region in order to form disulfide bonds among the cysteine residues, which was expected to increase the stability of the vaccine in the host body. This allowed the formation of disulfide bonds in the refined structure. Pairs of residues with appropriate geometries and the ability to form a disulfide bond were detected, and then the mutated model was designed by the DbD2 server (http://cptweb.cpt.wayne.edu/DbD2/ accessed on 10 March 2022) [64]. 

### 2.13. Codon Adaptations, In Silico Cloning, and Similarity Search with Host

For the purpose of cloning, the *E. coli* K12 strain was selected. Java Codon Adaptation Tool (JCAT, accessed on 12 March 2022) was the codon-adaptation tool utilized due to the dissimilarity between the codon usage of dogs and *E. coli*. During this action, restriction sites of BglII and Apa1, Rho-independent transcription termination, and prokaryote-ribosome-binding sites were avoided [65]. The optimized vaccine sequence was reversed, followed by the conjugation of the BglII cleavage site at the N-terminal and Apa1 cleavage site at the C-terminal. SnapGene tool was then used to set the adapted sequence into the pET28a (+) vector between the BglII (401) and Apa1 (1334). Finally, a similarity search with the host was performed using NCBI protein–protein Blast, in which a blast was performed against *Canis lupus* (Taxonomy ID: 9612).

## 3. Results

### 3.1. Identification of Protein Sequences and Antigenicity Screening

The twenty-one protein sequences of the VP2 gene of the canine parvovirus were systematically uploaded (Table 1) to the VaxiJen and TMHMM server on account of their possession of higher immunogenic potential. All the uploaded sequences were identified as antigenic and outside proteins. We also retrieved FASTA sequences of protein from the NCBI database. 

### 3.2. Identification of Homologous Protein Sets 

Four conserved areas of the VP2 protein were selected based on the analysis of their antigenic values (Table 2). The chosen conserved regions were longer than 15 nucleotides. These were utilized to predict T-cell and B-cell epitopes for vaccine design. 

### 3.3. T-Cell- and B-Cell-Epitope Prediction

The T-cell epitopes for the capsid protein VP2 that could bind to a significant proportion of DLA alleles were identified by analyzing the Immune Epitope Database (IEDB)’s MHC class-I binding predictions. The epitopes were selected based on their high binding affinity and their potential to interact with a large variety of DLAs. Promising candidates that were capable of eliciting T-cell responses were selected as prospective T-cell epitopes based on the TMHMM’s topological screening.

### 3.4. Superior Epitope Selection and Conservancy Prediction

Six T-cell epitopes (MHC-I-restricted) and four B-cell epitopes were selected based on high VaxiJen scores and lack of toxicity and allergenicity (Table 3). Overlapping sequences with higher antigenic scores were skipped during this skimming process. The conservancy analysis revealed the 100% conservancy of all the selected epitopes among the homologous strains.

### 3.5. Molecular Docking with Dog Leukocyte Antigen (DLA) Molecules

All the selected epitopes were docked with DLAs with a higher binding affinity (Table 4 and Table 5). Epitope “GTFYFDCKP” showed the highest binding affinity towards DLA-88*001:04, and epitope “GGTNFGYIG” was strongly bound to DLA-88*50801.

### 3.6. Vaccine Construction 

**Figure 2:**
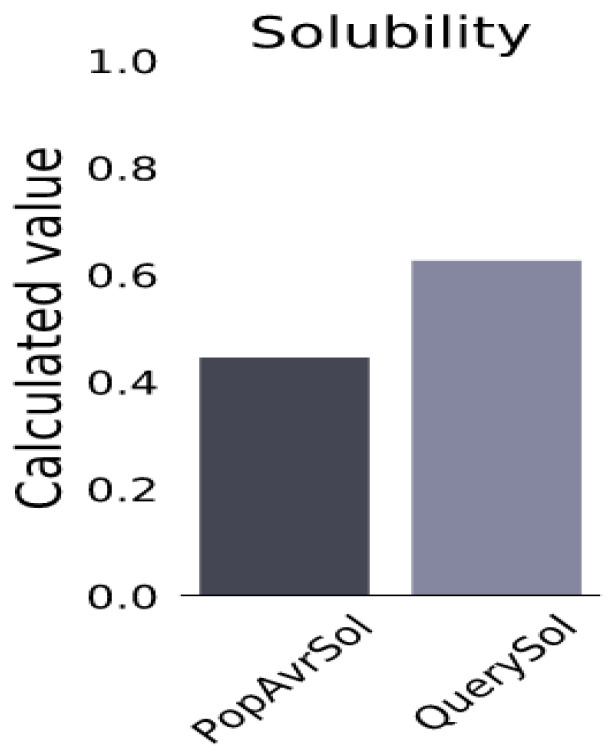
Solubility of constructed vaccine candidate, as obtained by protein-sol server. The solubility of the vaccine construct was found to be 0.627, and the population-average solubility was 0.45 in *E. coli*.

A fully fledged vaccine construct containing 272 amino acids was designed. The antigenic level was predicted to be higher than the threshold value, indicating its potential to increase immunogenicity in the body (Table 6). Moreover, it showed non-allergenic behavior after in silico prediction, and the solubility was 0.627, while the population average for the experimental dataset (PopAvrSol) was 0.45. Any scaled solubility value greater than 0.45 was predicted to have a higher solubility than the average soluble *E. coli* protein [66] (Figure 2).

### 3.7. Physico-Chemical Analyses of Vaccine Construct and Secondary Structure Prediction

**Figure 3:**
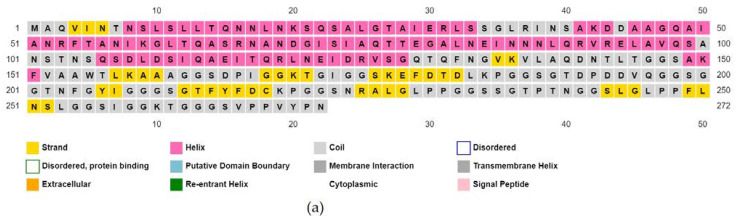

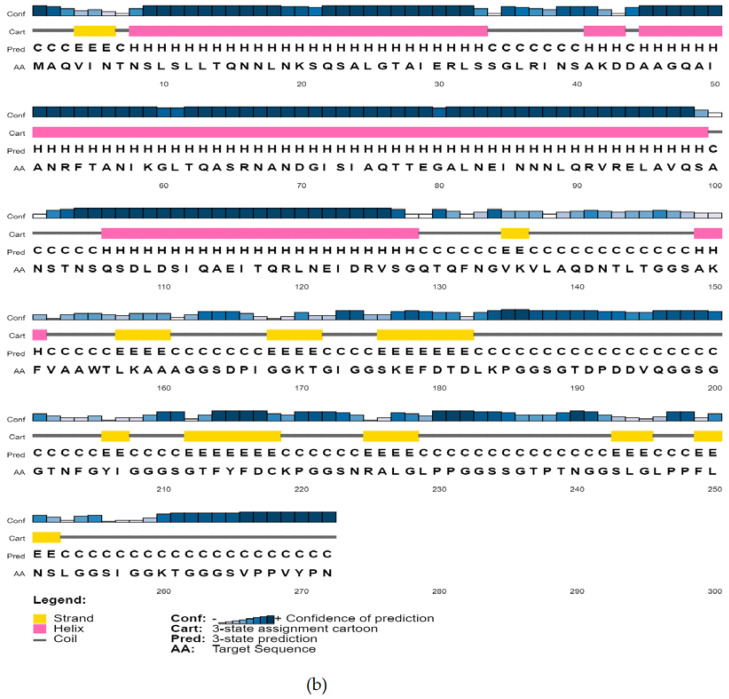
Predicted secondary conformation of the final vaccine construct by PSIPRED. (**a**) Sequence plot and (**b**) PSIPRED cartoon.

The molecular weight of the vaccine construct was 27,388.01 and was assumed to be stable in the body. Other properties of the vaccine molecule are listed in Table 6. The secondary structure predicted by PSIPRED is shown in Figure 3.

### 3.8. Tertiary-Structure Prediction 

**Figure 4:**
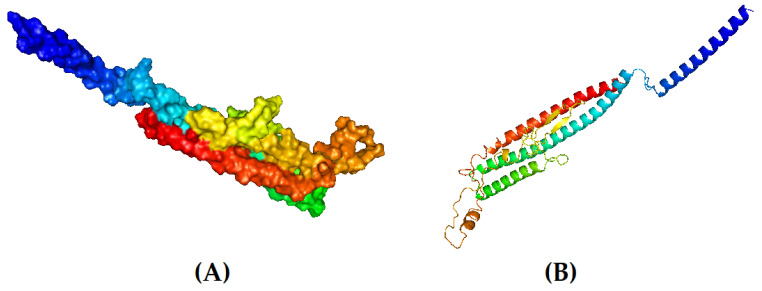
Tertiary structure of the designed vaccine candidate. (**A**) Surface view and (**B**) cartoon format.

**Figure 5:**
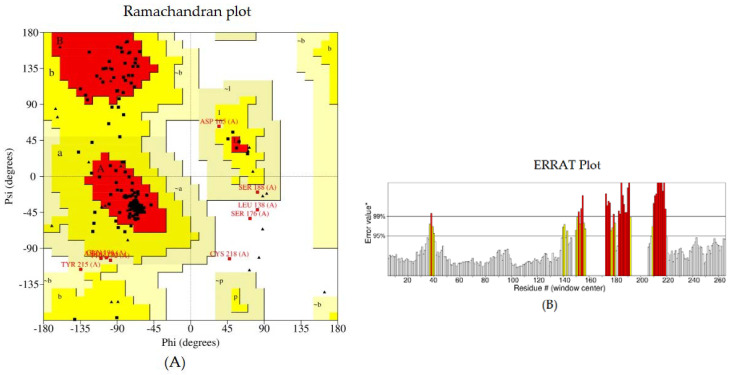
Structural validation of the designed vaccine candidate: (**A**) Ramachandran plot generated using PROCHECK. The areas showing different colors and letters, i.e., red (A, B, L), yellow (a, b, l, p), and light yellow (~a, ~b, ~l, ~p) represent the most favored regions, additional allowed regions, and generously allowed regions, respectively. Non-glycine residues are shown as squares, while glycine residues are shown as triangles. (**B**) The ERRAT plot.

The tertiary model of the refined structure is represented in Figure 4. The ERRAT score of the refined structure was 80.8696 and, in the Ramachandran plot, 85.8% of the residues were in the core region and 9.9% were in the allowed region (Figure 5). 

### 3.9. Binding Affinity and Stability of the Vaccine–TLR 5 Binding Complex

**Figure 6:**
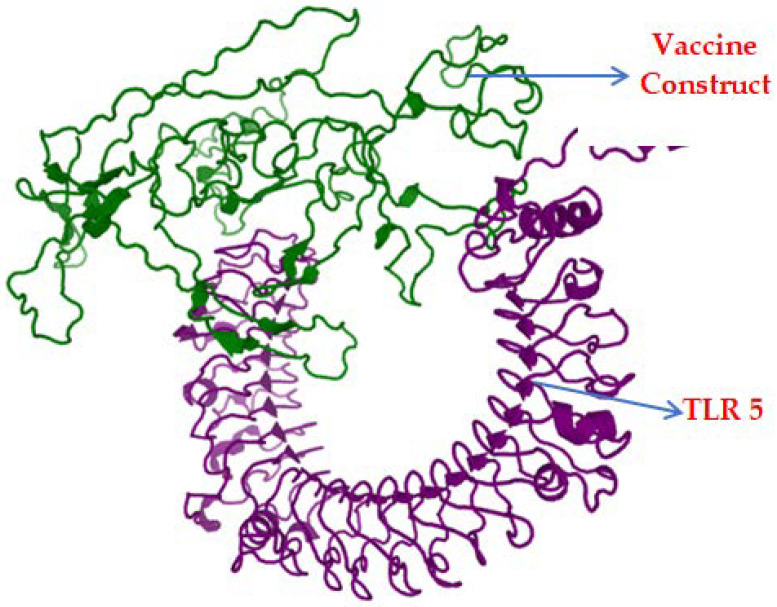
Docked complex of the vaccine construct (green) and TLR-5 (purple).

Binding affinity predicted by GalaxyTongDock was 1306.738, and the docked complex is represented in Figure 6.

**Figure 7:**
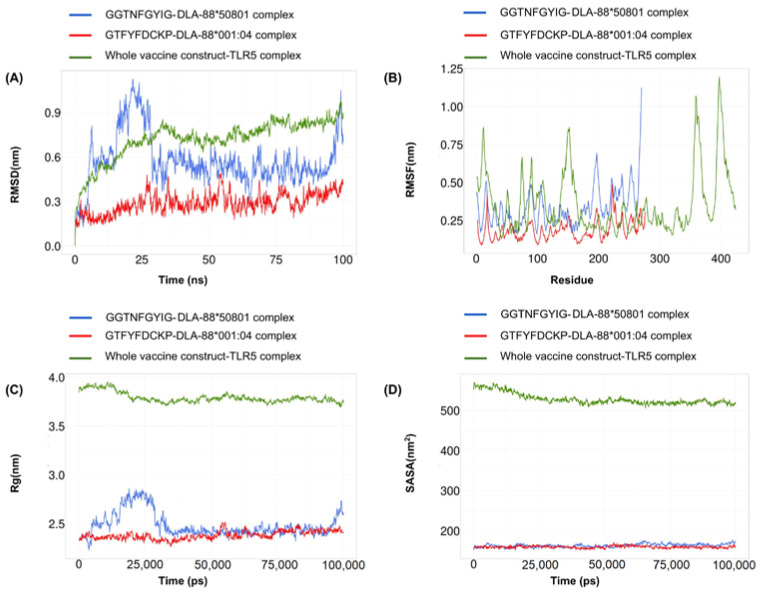
A 100-nanosecond molecular-dynamics simulation for the GTFYFDCKP-DLA-88*001:04 complex, GGTNFGYIG-DLA-88*50801 complex, and the whole vaccine construct–TLR5 complex. (**A**) RMSD of the C-alpha atoms during the simulation. (**B**) RMSF values of the alpha carbon during the simulation. (**C**) Radius of gyration (Rg) during the simulation. (**D**) SASA values during the simulation.

In the molecular-dynamics analysis, a root-mean-square deviation (RMSD) calculation was performed to evaluate the stability of the systems. The changes in the RMSD value correspond to the conformational changes of the protein as a result of the ligand binding. For the GTFYFDCKP-DLA-88*001:04 complex, the RMSD value ranged between 0.2 and 0.4. In case of the GGTNFGYIG-DLA-88*50801 complex, a large RMSD value was observed between 0 and 25 ns. However, it remained relatively stable for the rest of the simulation. For the vaccine construct, no drastic alterations in the RMSD profile were observed (Figure 7A).

Root-mean-square fluctuation (RMSF) was used to determine the regional flexibility of the protein. The higher the RMSF, the greater the flexibility of a given amino acid position. Both the GTFYFDCKP-DLA-88*001:04 and the GGTNFGYIG-DLA-88*50801 complexes showed similar RMSF profiles, but there was a sharp peak near the end of the DLA-88*50801. For the vaccine construct, there were several patches of highly flexible regions, such as one at the beginning and two near the end of the vaccine (Figure 7B).

The radius of gyration (Rg) is a measure to determine the degree of compactness. A radius of gyration with a relatively stable value means the stable folding of a protein. Fluctuation in the radius of gyration implies the unfolding of the protein. The radius-of-gyration analysis indicated that the GGTNFGYIG-DLA-88*50801 complex was more compact than the GTFYFDCKP-DLA-88*001:04 complex. The vaccine construct gradually reached compactness during the course of the 100-nanosecond simulation (Figure 7C).

The solvent-accessible surface area (SASA) was used in the MD simulations to predict the hydrophobic core stability of the proteins. The higher the SASA value, the higher the chance of destabilization of the protein due to solvent accessibility. The SASA values for the GTFYFDCKP-DLA-88*001:04 and the GGTNFGYIG-DLA-88*50801 complexes showed few differences. In case of the vaccine construct, the SASA value decreased to very low levels near the end of the simulation (Figure 7D).

### 3.10. Disulfide Engineering of the Vaccine Construct

**Figure 8:**
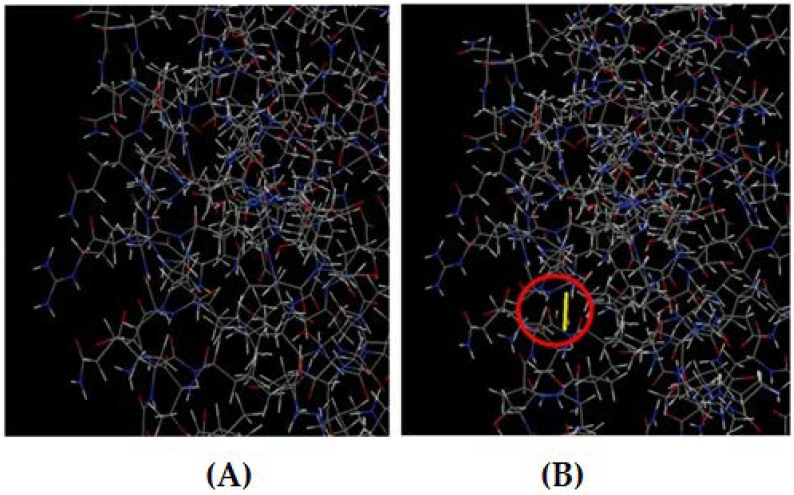
(**A**) Original 3D model. (**B**) Mutant model with a disulfide bond (indicated by the red circle).

The DbD2 server revealed a total of 26 amino acid pairs that have the potential to form disulfide bonds. Only two pairs (Phe216-Gly222 and Phe179-Ser232) could be replaced with cysteine, as they were found to be suitable for the formation of disulfide bonds based on the energy, chi3, and B-factor parameters. In residue screening, a chi3 value between −87 and +97 and energy < 2.5 were considered. The mutant model was then built by replacing all these residues with a cysteine residue (Figure 8). 

### 3.11. Codon Adaptation, In Silico Cloning, and Similarity Search with Host

**Figure 9:**
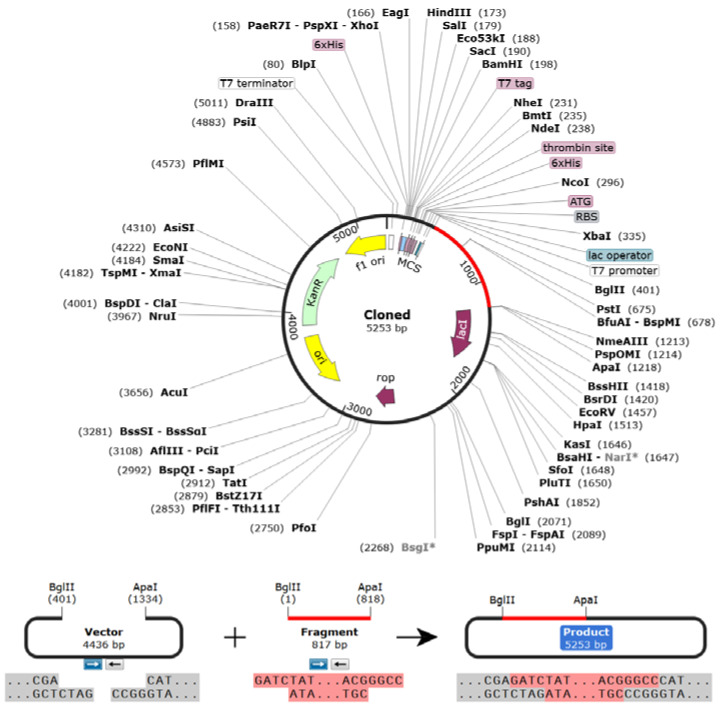
Vaccine constructs in the pET28a (+) vector. During cloning, the codon-optimized vaccine sequence was inserted between BglII (401) and ApaI (1218) restriction sites of the pET28a (+) vector (vaccine construct is shown in magenta color).

The Codon Adaptation Index (CAI) was predicted to be 0.98, indicating the higher proportion of most of the abundant codons. The GC content of the adapted sequence was 53.78, indicating a significant increase in GC content. Moreover, the sequence did not contain any cleavage sites of BglII or ApaI, indicating its safety during cloning. Following the SnapGene cloning, a clone of 5253 bp with an insert size of 817 bp was created (Figure 9). Finally, the NCBI blast revealed no sequence similarities between the vaccine construct and the host. 

## 4. Discussion

Viruses in different species can have unexpected connections with one another. For example, CPV-2 is believed to have evolved from mutations in FPV through adaptation in the canine host [11,12,13]. Through cross-species transmission, CPV has evolved with the characteristics of a broad host range, host-specific adaptation, and possible pandemic spread [67,68]. This is because genetic mutations and the deletion or rearrangement of key sites can change the host range and pathogenicity. As an example, viral mutants, such as an FPV mutant, have been isolated from primates [69]. Similarly, the CPV-2 can continue to mutate, and humans can eventually become infected. Certain significant infectious diseases, such as severe acute respiratory syndrome (SARS), highly pathogenic avian influenza (H5N1, H1N1), Nipah, and Ebola, originate in non-human hosts, but can cause severe outbreaks in the human population [70]. 

In recent decades, the infectious canine parvovirus has emerged as a serious invisible enemy of dogs worldwide, undergoing multiple mutations and expanding the host range [71]. Changes in gut mucosal architecture and the microbial dysbiosis of the gut microbiota have been associated with the severity of CPV infection [72]. In such cases, the mucosal barrier separating gut microbes from the bloodstream might be disrupted, and the microbes might enter the bloodstream, causing systemic inflammatory responses [73]. The therapeutic management of CPV-infected dogs using various antibiotics also raises concerns about antimicrobial resistance (AMR), which is a major public health concern in all countries [73]. Vaccination is an effective intervention to reduce this burden. However, conventional vaccines may have some limitations; for example, the virulence of an attenuated virus may be reversed, or the inactivation process may not suppress the virulence [74,75]. In addition, the development of such vaccines usually requires large budgets and lengthy timelines. To avoid such constraints, the development of multi-epitope-peptide vaccines containing protective epitopes has been an area of focus [76]. Peptide vaccines can be designed to elicit B-cell and T-cell responses, and computational approaches can be used to predict candidate epitopes, making the development of such vaccines both safe and inexpensive [77,78]. 

Considering that the outer VP2 protein is exposed outside of the virion and, hence, may be a viable target for T-cell- and B-cell-epitope discovery [79], the focus of this study was on the sequences in this area. Using computational methods, we evaluated the physicochemical properties and antigenic potential of the VP2-viral-protein sequences. As a structural protein candidate, VP2 has high immunogenicity [80].

Structural proteins are the first targets for generating epitope-based peptide vaccines. Epitopes on viral particles interact with cell receptors, influencing disease pathogenesis [81]. Monovalent vaccines target a single pathogen or organism to elicit immunogenic responses [82]. These responses include B-cell-mediated antibody responses and cytotoxic CD8 T lymphocyte (CTL) responses, which can identify and destroy foreign antigens [83]. The design of T-cell-epitope-based peptide vaccines has been successfully demonstrated in the past against the SARS CoV-2 [84], Banna virus [76], and Zika virus [30]. 

We performed a homology blast of the selected protein sequences in NCBI to collect homologous sets of the protein in various strains of the virus. This was followed by multiple sequence alignments to obtain similar protein fragments among those proteins. This was undertaken to ensure the effectiveness of the vaccine against all the strains of the virus. The MHC-I-binding-prediction tool at IEDB helped to forecast the presence of several immunogenic VP2 epitopes, each of which binds to a high proportion of DLA alleles with a binding affinity that affects and adjusts the T-cell response. The DLA-allele specificity of T-cell epitopes can be a determinant for generating appropriate immunological responses [85]. Putative T-cell epitopes were selected based on their association with the highest numbers of DLA alleles, outer topology, and antigenic score.

The selected epitopes were docked with the DLAs with the highest binding affinity. Epitopes “GTFYFDCKP” and “GGTNFGYIG” showed the highest binding affinities to DLA-88*001:04 and DLA-88*50801, respectively. The B-cell epitopes were predicted using different algorithms, such as Bepipred Linear Epitope Prediction 2.0 [42], Emini surface-accessibility prediction [43], and the Kolaskar-and-Tongaonkar antigenicity scale [44], to assess the ease of their accessibility and antigenicity. The antigenic score was used to select the best epitopes. All the selected T-cell and B-cell epitopes exhibited 100% conservancy among all the proteins of the homologous protein sets. 

A fully fledged vaccine construct containing 272 amino acids was constructed by combining the best epitopes linked together via a suitable linker, with adjuvant and PADRE sequences. The currently used GGS linker was previously used to develop multi-epitope vaccines against the herpes simplex virus [86], *candida auris* [23], dengue virus [29], and canine circovirus [62]. Linkers have been found to ensure the efficient separation of individual epitopes in vivo [87,88,89,90]. The PADRE sequence helped to mitigate the problem caused by the highly polymorphic DLA alleles. Vaccine constructs containing PADRE sequences led to better CTL responses [91]. 

Before the tertiary-structure prediction and 3D model refinement, the physicochemical properties and solubility of the predicted vaccine were investigated. The molecular weight of the vaccine construct was 27.38 kDa. Any vaccine with a molecular weight < 110 kDa is considered a viable target for vaccine development due to its easier and faster expression and rapid purification [92,93]. The aliphatic index of the vaccine was calculated as 73.97, indicating that the vaccine is stable over a wide temperature range [94]. The theoretical pI of the proposed vaccine was set at 5.51, suggesting that the vaccine has acidic properties and is similar to those of MEV candidates against fatal visceral leishmaniasis [95]. The GRAVY score was calculated as −0.388; this negative value indicates that the vaccine interacts better with water molecules [96,97]. The vaccine-instability index was 30.77, which is less than 40, indicating that the vaccine candidate is stable under standard conditions [55]. The half-life of our vaccine candidates was estimated to be more than 20 h in yeast and more than 10 h in *E. coli*, indicating that the vaccine was exposed to the immune system for a longer period of time [98]. Moreover, in terms of solubility, the vaccine developed in the present study had a good solubility value (0.627), which was above the threshold value (0.45), and suggests that it is an ideal vaccine candidate [99]. In addition, the analysis revealed that the vaccine construct is non-allergic, highly antigenic, and non-toxic to the host. 

In addition, the secondary structure of the predicted novel vaccine candidate exhibited a higher coil structure, which could play an important role in the high level of flexibility of proteins and in the enhancement of the antibody-binding ability [98]. The ERRAT score of the refined structure was 80.8696, and in the Ramachandran plot, 85.8% of the residues were in the core region and 9.9% were in the allowable region. These values indicated that the predicted structure may be considered a promising vaccine candidate. The molecular docking and molecular-dynamics simulations were also performed to evaluate the vaccine’s interactions with the canine TLR5 receptor, and the predicted vaccine was found to be both flexible and stable. The CAI value of our vaccine (0.98) and the GC content (53.78) were also within the optimal range, potentially indicating higher expression in the *E. coli* K-12 system [100]. Finally, the codon adaptation and in silico cloning indicated that the vaccine was suitable for large-scale production in *E. coli* [23]. Future studies will include further experimentation using animal models for the predicted vaccine.

## 5. Conclusions

In the present study, a novel multi-epitope vaccine against canine parvovirus integrating superior T-cell and B-cell epitopes was designed using immunoinformatics approaches. The vaccine construct was evaluated as highly antigenic and immunogenic, and it can elicit potent immune responses. Additionally, the vaccine was found to be safe, stable, non-allergenic, and non-toxic, with good physicochemical properties, ensuring that it can strongly interact with canine TLR-5. The results of this study are promising; however, further experiments through in vitro and in vivo studies should be performed to confirm the reliability, efficacy, and safety of the vaccine constructs. 

## Figures and Tables

**Table 1:** Topologies and VaxiJen scores of sequences of the VP2 protein of canine-derived CPV-2.

Sl. No.	Sequence ID	Topology	VaxiJen Score
1	DPHSAUBD11N001	Outside	0.5468
2	DPHSAUBD11N008	Outside	0.5263
3	DPHSAUBD11N013	Outside	0.5263
4	DPHSAUBD12N042	Outside	0.5381
5	DPHSAUBD12N044	Outside	0.4930
6	DPHSAUBD12N067	Outside	0.4933
7	DPHSAUBD13N069	Outside	0.5294
8	DPHSAUBD13N073	Outside	0.5263
9	DPHSAUBD13N081	Outside	0.5386
10	DPHSAUBD13N100	Outside	0.5477
11	DPHSAUBD21N118	Outside	0.5180
12	DPHSAUBD21N125	Outside	0.5209
13	DPHSAUBD21N147	Outside	0.5289
14	DPHSAUBD22N163	Outside	0.5209
15	DPHSAUBD12N056	Outside	0.5285
16	DPHSAUBD22N169	Outside	0.5209
17	DPHSAUBD22N170	Outside	0.5310
18	DPHSAUBD22N196	Outside	0.5310
19	DPHSAUBD23N200	Outside	0.5285
20	DPHSAUBD23N213	Outside	0.5334
21	DPHSAUBD23M249	Outside	0.4976

**Table 2:** Conserved regions of the VP2 protein.

Sequence	Topology	VaxiJen Score
SGTPTNIYHGTDPDDVQ	Outside	0.6334
EFATGTFYFDCKPCRLTHTWQTNRALGLPPFLNSLPQAEGGTNFGYIG	Outside	0.7204
LLPTDPIGGKTGINYTN	Outside	0.8635
VPPVYPNGQIWDKEFDTDLKP	Outside	0.4321

**Table 3:** Predicted superior epitopes and their immunogenic properties *.

Sl. No.	EPITOPE	VaxiJen Score	Allergenicity	Toxicity	Epitope Length	Conservancy
1	DPIGGKTGI	0.9259	Non-allergenic	Non-Toxic	9	100.00%
2	KEFDTDLKP	0.9968	Non-allergenic	Non-Toxic	9	100.00%
3	GTDPDDVQ	0.9231	Non-allergenic	Non-Toxic	8	100.00%
4	GGTNFGYIG	1.9328	Non-allergenic	Non-Toxic	9	100.00%
5	GTFYFDCKP	1.5199	Non-allergenic	Non-Toxic	9	100.00%
6	NRALGLPP	1.3745	Non-allergenic	Non-Toxic	8	100.00%
7	SGTPTN	0.9542	Non-allergenic	Non-Toxic	6	100.00%
8	LGLPPFLNSL	0.4422	Non-allergenic	Non-Toxic	10	100.00%
9	IGGKTG	1.5579	Non-allergenic	Non-Toxic	6	100.00%
10	VPPVYPN	0.9901	Non-allergenic	Non-Toxic	7	100.00%

* Epitopes 1 through 6 are T-cell epitopes, while epitopes 7 through 10 are B-cell epitopes.

**Table 4:** Docking scores of T-cell epitopes (MHC I-restricted) with DLA-88*001:04 (7CJQ).

Epitope	Global Energy	Hydrogen Bond Energy	ACE *	Score	Area
DPIGGKTGI	−40.88	−1.45	−2.66	7456	839.80
KEFDTDLKP	−4.44	0.00	−1.05	7506	1012.50
GTDPDDVQ	−14.84	−1.82	1.17	6786	852.50
GGTNFGYIG	−50.33	−5.65	−8.81	7152	903.70
GTFYFDCKP	−55.54	−1.05	−9.60	8484	81,119.50
NRALGLPP	−14.84	−2.37	6.23	6910	846.30

* Atomic contact energy (ACE) is the desolvation free energy required to transfer atoms from water to a protein’s interior [52].

**Table 5:** Docking scores of T-cell epitopes (MHC I-restricted) with DLA-88*50801 (5F1I).

Epitope	Global Energy	Hydrogen Bond Energy	ACE	Score	Area
DPIGGKTGI	−28.98	−0.50	−2.00	7650	979.60
KEFDTDLKP	−24.03	−2.90	7.58	8850	1026.00
GTDPDDVQ	−29.84	−3.54	0.50	7080	826.60
GGTNFGYIG	−49.95	−2.71	−7.60	7516	895.60
GTFYFDCKP	−30.79	−2.33	−5.91	9338	1134.70
NRALGLPP	−36.67	−2.58	−4.98	7576	979.10

**Table 6:** Amino acid sequences and properties of constructed vaccine.

Vaccine Construct	Properties	Value
MAQVINTNSLSLLTQNNLNKSQSALGTAIERLSSGLRINSAKDDAAGQAIANRFTANIKGLTQASRNANDGISIAQTTEGALNEINNNLQRVRELAVQSANSTNSQSDLDSIQAEITQRLNEIDRVSGQTQFNGVKVLAQDNTLTGGSAKFVAAWTLKAAAGGSDPIGGKTGIGGSKEFDTDLKPGGSGTDPDDVQGGSGGTNFGYIGGGSGTFYFDCKPGGSNRALGLPPGGSSGTPTNGGSLGLPPFLNSLGGSIGGKTGGGSVPPVYPN	Length	272
	Molecular weight	27,388.01
	Aliphatic index	73.97
	Theoretical pI	5.51
	GRAVY value	−0.388
	Instability index	30.77 (stable)
	Extinction coefficients	9970
	Estimated half-life	>20 h (yeast, in vivo) >10 h (*Escherichia coli*, in vivo)
	Antigenicity	0.5696 (Probable ANTIGEN)
	Solubility	0.627
	Allergenicity	Probable NON-ALLERGEN

## Data Availability

All relevant data are included within the manuscript.
